# The first OSCE; does students’ experience of performing in public affect their results?

**DOI:** 10.1186/s12909-015-0343-0

**Published:** 2015-03-26

**Authors:** Michael Chan, Nigel Bax, Caroline Woodley, Michael Jennings, Rod Nicolson, Philip Chan

**Affiliations:** 1 Bart’s and the London Medical School, London, UK; 2 University of Sheffield Medical School, Sheffield, UK; 3 Department of Psychology, University of Sheffield, Sheffield, UK

**Keywords:** OSCE, Performance anxiety, Personality, Admissions statement

## Abstract

**Background:**

Personal qualities have been shown to affect students’ exam results. We studied the effect of experience, and level, of public performance in music, drama, dance, sport, and debate at the time of admission to medical school as a predictor of student achievement in their first objective structured clinical examination (OSCE).

**Methods:**

A single medical school cohort (n = 265) sitting their first clinical exam in 2011 as third year students were studied. Pre-admission statements made at the time of application were coded for their stated achievements in the level of public performance; participation in each activity was scored 0–3, where 0 was no record, 1 = leisure time activity, 2 = activity at school or local level, 3 = activity at district, regional or national level. These scores were correlated to OSCE results by linear regression and *t*-test. Comparison was made between the highest scoring students in each area, and students scoring zero by *t*-test.

**Results:**

There was a bell shaped distribution in public performance score in this cohort. There was no significant linear regression relationship between OSCE results and overall performance score, or between any subgroups. There was a significant difference between students with high scores in theatre, debate and vocal music areas, grouped together as verbal performance, and students scoring zero in these areas. (p < 0.05, *t*-test) with an effect size of 0.4.

**Conclusions:**

We found modest effects from pre-admission experience of verbal performance on students’ scores in the OSCE examination. As these data are taken from students’ admission statements, we call into question the received wisdom that such statements are unreliable.

## Background

Judgement of the personal qualities of students applying for admission to medical school is difficult. It is acknowledged that this is a different dimension to students’ academic qualities, and that it is relevant to the selection of medical professionals. Furthermore, longitudinal studies show that some personal qualities, measured in various ways at the time of admission, have predictive validity: that is, these qualities correlate with later student and professional achievement [1,2].

One of the recurring themes we have seen in students who have struggled with their first exposure to the objective structured clinical examination, or OSCE, has been a form of “stage fright” or performance anxiety (we shall henceforward use the term “performance” in this context, rather than meaning some measure of students’ results and achievements). This is supported by Mavis, who administered a self-efficacy survey to students ten minutes before their OSCE examination, and found that their subsequent attainment was “the product of complex relationships between skills and knowledge, mediated by students perceptions of anxiety, self-confidence and preparedness” [3]. Sarid studied students sitting different types of examinations, and found that OSCEs induced a greater depression/dejection reaction afterwards compared with oral and written exams [4]. Dental students perceived OSCEs as the most stressful of all their assessment modalities [5].

Performance anxiety is to some extent dependent on personality, but could be mitigated with exposure to the situation [6]. However, as students had no prior exposure to OSCEs (this was their first one), we considered that training and experience would be gained only in environments outside of OSCEs. The face to face nature of the assessment and the pressure of performance would most likely be experienced during public performances in the students’ past, and related to extracurricular, rather than academic, activities. The research question that followed these observations is whether experience of performance in public before the OSCE would affect students’ results.

Urlings-Strop and colleagues compared the performance of students selected according to the quality of their extracurricular activities as well as cognitive tests to a group who were allocated to the school by the Netherlands lottery [2]. They found a significant difference in medical school performance favouring the selected group. Among the selection criteria were extracurricular performance in sports, music or science as well as work experience and roles in management and organization. They advanced an explanation that skills involved with extracurricular activities at the time of admission might generically be translatable to high academic achievement later in medical school. Possibly a significant part of those skills might be the ability to perform in public.

## Methods

### Sample

An entire year cohort (n = 265) sitting their first-ever OSCE as third year students was studied. Third year students start their clinical course (third year) in July of the same year, and have had 12 weeks’ clinical placements on hospital wards before the OSCE. This was a high stakes examination, with a pass grade needed for progression. Failure would mean re-sitting another OSCE some weeks later and a second failure at re-sit would necessitate repeating the year, or being excluded from the course, depending on previous academic record. All students took the examination on the same day under secure conditions. The exam was comprised of 12 stations, 6 of which were history-taking from trained simulated patients, and 6 were physical examination on either real patients or models. Some history stations had two examiners, one assessing the basic medical skill, and the other assessing communication skill. All other stations had single examiners. The mark schemes included discretionary marks for a logical, well-ordered history or examination, which could count as many as 4 marks out of a total of 25 for each station. OSCE results were analysed as overall results, and separately as results from history stations, and physical examination stations.

Pre-admission statements made at the time of application (UCAS personal statements) were available in individual student files. Following ethics committee approval (Project SMBRER232), these statements were coded by a researcher (MC) who was not a student or graduate of this university. This was a specific requirement of the ethics committee.

### Measures

The statements were examined for evidence of public performances that would accustom the participant to the experience of performance anxiety. We reasoned that the amount of anxiety would rise with the setting of the performance, and that if the stakes were high, anxiety would be high and students more accustomed to coping with this. We coded participation in vocal and instrumental music, theatre, dance, sport and debate/public speaking; we grouped together vocal music, theatre and debate/public speaking as verbal activities, and sport, instrumental music and dance as motor activities.

Participation in each activity was scored 0–3, where 0 was no record, 1 = leisure time activity, 2 = activity at school or local level, 3 = activity at district, regional or national level. Sample statements were considered by a group of senior staff who were the Director of Teaching, the admissions tutor, and the head of third year (NB, MJ, CW), and agreement reached for scoring various levels of performance. A single coder then applied this framework to the whole student cohort. Doubtful cases were taken to these senior staff for guidance. A random sample of coded statements were reviewed to check for agreement and consistency.

Scores in each activity were used for analysis. Scores in verbal and motor groups were derived as the sum of all scores for activities in these categories; total performance score was the sum of all activity scores.

### Analysis

We thought there may be a correlation between verbal performance and history OSCE score and motor performance and physical examination OSCE scores. Therefore we correlated performance scores with OSCE results in two ways; first, all scores were correlated with all results; and second, the extreme groups method was used [7], correlating the groups scoring highest and lowest for each activity with their results. For a single activity, this compared the group scoring 2 or 3 with the group scoring 0. Students scoring 1 represented the median for all activities, and were not included in the extreme groups analyses. For the composite verbal and motor activity scores, the group of students scoring >3 (verbal) or >5 (motor) were compared with the zero group. These scores corresponded to the around the top 10% of scores in these groups. Scores were correlated to OSCE results by Pearson product moment correlation, linear regression, *t*-test and Chi-square as relevant.

Effect size of differences in each activity was calculated by standard methods, (Mean_top scores_- Mean_zero scores_)/S.D._both populations_. Effect sizes of 0.4 or greater are conventionally regarded as large in the educational literature [8].

### Ethics

This study was granted ethical approval by the University of Sheffield Medical School Ethics Review panel, ref SMBRER232.

## Results

**Figure 1:**
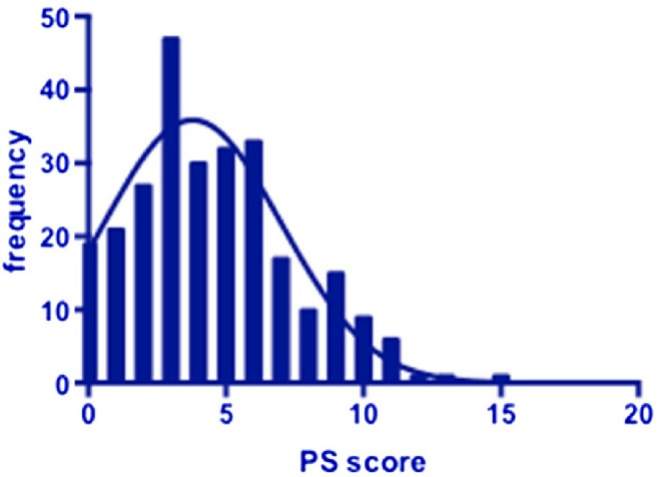
**Frequency histogram showing range of total performance scores (maximum possible score = 18) for the student cohort.**

There was a variation for total scores in public performance activities in this cohort, ranging from 0–15 (Figure 1).

**Figure 2:**
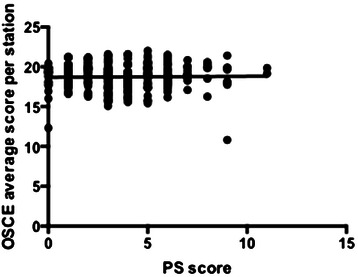
**Linear regression of total performance score (PS) correlated to OSCE score for the student cohort.**

There was no linear correlation between total scores in public performance activities and the overall OSCE results, r = 0.00035 (Figure 2).

**Figure 3:**
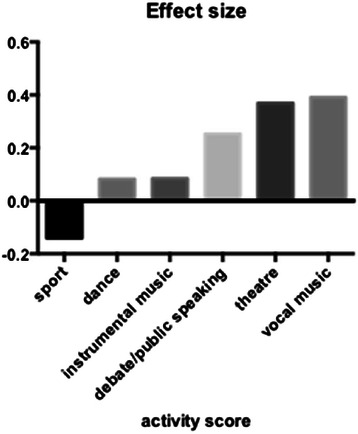
**Extreme groups comparison.** Effect size of top scores (3) against low scores (0) for individual activities.

There were both positive and negative effects of high scores in various individual activities on OSCE results, but total numbers were often low, and therefore no single activity was statistically significant (Figure 3). The composite group of verbal activities did show statistically significant difference between extreme groups, p < 0.05 (*t*-test). Verbal activity high scores were significantly related to better history and better examination station results in the OSCE. There was a non-significant negative effect for motor activities. There is a significantly increased chance of passing the OSCE if the verbal score >3, compared to a significantly increased chance of achieving borderline/fail grade if the verbal score is zero, (Chi square, Yates correction, one tail p = 0.045.) 55 students who had verbal activity scores >3 passed the OSCE but only 7 such students failed. Conversely, of a total of 59 students who were borderline or failed, 44 had scored zero for verbal activities (75%); of 194 students who passed, 120 scored zero for verbal activities (62%)

.

**Figure 4:**
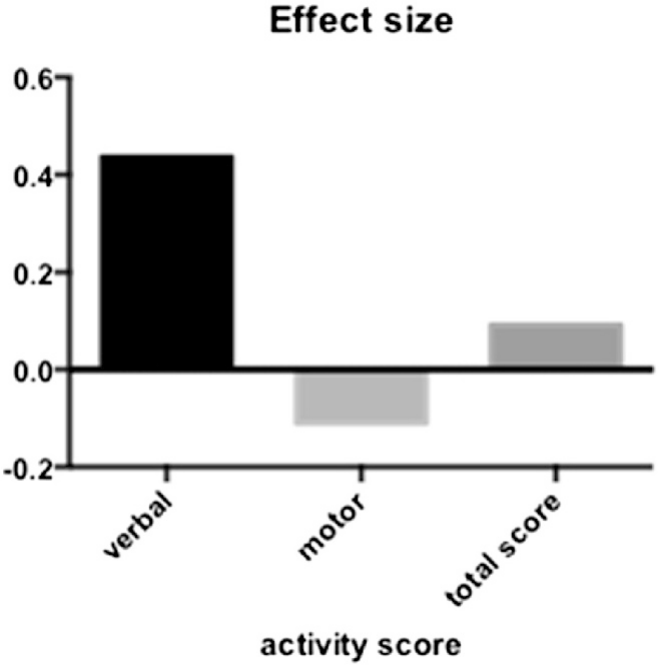
**Extreme groups comparison.** Effect size of activities grouped as verbal (comparing aggregate score ≥ 3 to zero) or motor (comparing aggregate score ≥ 5 to zero) on OSCE results.

The effect size of verbal activities on OSCE results is calculated at 0.44. The corresponding effect size for motor activities is −0.11 (Figure 4). We did not observe differential effects on history and examination OSCE stations.

## Discussion

We found a contrast between straightforward linear regression and the extreme groups approach in terms of relating performance experience to OSCE results. Naturally, OSCE results are not the product of performance alone, and also test knowledge, experience, preparedness and self-efficacy [3]. We argue that the extreme groups approach is actually more relevant in this analysis than the all-comers approach; that the effect size of the public performance variable is likely only to be revealed at the extremes rather than in the median groups due to its interaction with many other significant variables. We would not expect the self-declared statement of extracurricular activities to predict exam results with greater power. We consider that, as students who do poorly in the first OSCE actually tend to show declining OSCE results in the future, this justifies the extreme groups approach [9].

There is at least a three year gap between the admission statement and the OSCE. For young undergraduates in their early twenties, this is a significant time gap, and many things would have changed. Nevertheless, if the experience existed and was declared in their admission statement, it would not have disappeared in the intervening time. We acknowledge that students may not have declared their extracurricular activities fully in the admission statement or have developed new activities in the interim. However, this direction of error would have the effect of minimizing the differences between high and low scorers; in the opposite direction to our results; so paradoxically strengthening our findings. We were also impressed by the study of Urlings-Strop and colleagues cited above [2], who used extracurricular activities at admission to select a group of students who subsequently showed high performance in clinical clerkships, around 4–5 years later.

We wanted to use a measurement of experience of public performance that was relevant to students sitting their first OSCE. We decided to use student’s personal statements, which were made at the time of application to medical school. These statements were produced to maximize their chances of admission, so we felt that they were unlikely to miss out their achievements in extracurricular activities. Some of these extracurricular activities involved performing in public, and were directly relevant to our purpose. We could have obtained some of this information by questionnaire during the third year, but we would not have obtained complete information on the whole cohort. Statements in either admission statements or questionnaire responses cannot be directly verified.

The personal statement in the UCAS application is regarded as lacking in predictive validity. We believe we have some additional insight into this belief. Some previous studies have all looked at a global rating for the whole statement, rather than analyzing specific data from within the statement [10,11]. This would agree with our experience with total performance score, which shows no relationship with OSCE results. However, this null result might have been an artefact, if the various components of the global ratings had both positive and negative correlations that cancelled each other out. We observed some negative associations, which would support this interpretation.

We assumed that participation in high-stakes performance activities would reduce performance anxiety. There is some data to support this; for example, it has been documented that just taking part in public speaking reduces subsequent anxiety in this area. However, performance anxiety can occur, even in professional musicians, who only perform in high-stakes situations [12,13].

Ferguson & et al. [14] performed content analysis of the UCAS statements of 176 students at the University of Nottingham medical school and obtained many different categories, coded as I (interest in) or C (contributes to the community) corresponding to our scores of 1 and 2/3. They went on to show lack of correlation of any of these categories with first year assessments of these students. Their themes included sports, playing musical instruments and choir/orchestra, as well as many more general ones such as voluntary work, illness in the family, liking travelling, and attending medical conferences. The important differences between our study and Ferguson are that we looked only at activities with significant public performance content, and our criterion was the third year OSCE. We also used the extreme groups approach, which magnifies the predictive effect size. Although we agree with Ferguson that there is no overall predictive validity of the activities declared in the admission statement, we were able to see the effect in some of the individual activities.

We acknowledge that our study only covered a single cohort of students at a single medical school, although numbers (265) are relatively large. We can therefore only justify limited conclusions; that there is modest evidence for the positive effect of students’ previous experience of intensive performance anxiety during the first OSCE. If the effect were stronger, or backed in a larger study, there could be a case made for including more public performance elements in the early medical school curriculum, or alternatively, for selection of students based partly on these relatively objective criteria [2,15].

We also recognize that these intensive public performance experiences are important not only in terms of examination prediction, but also stand as desirable qualities in their own right. Astin advanced the hypothesis that student involvement in co-curricular activities contributed to academic, cognitive and personal growth [16]. It is possible that experience of performance activities is a subset of a larger character trait, such as the tendency to be involved in things, or even the ability to manage time effectively, either of which might be a predictor of overall performance in higher education [17]. However, the most obvious and parsimonious explanation is that experience in high-stakes performance mitigates performance anxiety. Nevertheless, performance in public is only one of many possible desirable traits in medical students. Some of them are intrinsically desirable, and do not require predictive validity [18].

## Conclusions

There is a modest effect of previous experience of public performance and achievement in the first OSCE in a cohort of medical students at a single school. As the data on public performance was derived from application statements, this also represents a predictive validity from a particular component of the application statement.
